# Synergies of Human Umbilical Vein Endothelial Cell-Laden Calcium Silicate-Activated Gelatin Methacrylate for Accelerating 3D Human Dental Pulp Stem Cell Differentiation for Endodontic Regeneration

**DOI:** 10.3390/polym13193301

**Published:** 2021-09-27

**Authors:** Wei-Yun Lai, Tzu-Hsin Lee, Jian-Xun Chen, Hooi-Yee Ng, Tsui-Hsien Huang, Ming-You Shie

**Affiliations:** 1 School of Dentistry, Chung Shan Medical University, Taichung 406040, Taiwan; joe1112joe@hotmail.com; 2 Department of Orthodontics, Changhua Christian Hospital, Changhua 500, Taiwan; cheryb22@gmail.com; 3 School of Medicine, China Medical University, Taichung 40447, Taiwan; d21996cmuh@gmail.com (J.-X.C.); hooiyeen@gmail.com (H.-Y.N.); 4 Department of Surgery, China Medical University Hospital, Taichung 406040, Taiwan; 5 x-Dimension Center for Medical Research and Translation, China Medical University Hospital, Taichung 40447, Taiwan; 6 Department of Stomatology, Chung Shan Medical University Hospital, Taichung 40201, Taiwan; 7 School of Dentistry, China Medical University, Taichung 40447, Taiwan; 8 Department of Bioinformatics and Medical Engineering, Asia University, Taichung 41354, Taiwan

**Keywords:** calcium silicate, gelatin methacryloyl, odontogenesis, dental pulp stem cell, human umbilical vein endothelial cell

## Abstract

According to the Centers for Disease Control and Prevention, tooth caries is a common problem affecting 9 out of every 10 adults worldwide. Dentin regeneration has since become one of the pressing issues in dentistry with tissue engineering emerging as a potential solution for enhancing dentin regeneration. In this study, we fabricated cell blocks with human dental pulp stem cells (hDPSCs)-laden alginate/fish gelatin hydrogels (Alg/FGel) at the center of the cell block and human umbilical vascular endothelial cells (HUVEC)-laden Si ion-infused fish gelatin methacrylate (FGelMa) at the periphery of the cell block. ^1^H NMR and FTIR results showed the successful fabrication of Alg/FGel and FGelMa. In addition, Si ions in the FGelMa were noted to be bonded via covalent bonds and the increased number of covalent bonds led to an increase in mechanical properties and improved degradation of FGelMa. The Si-containing FGelMa was able to release Si ions, which subsequently significantly not only enhanced the expressions of angiogenic-related protein, but also secreted some cytokines to regulate odontogenesis. Further immunofluorescence results indicated that the cell blocks allowed interactions between the HUVEC and hDPSCs, and taken together, were able to enhance odontogenic-related markers’ expression, such as alkaline phosphatase (ALP), dentin matrix phosphoprotein-1 (DMP-1), and osteocalcin (OC). Subsequent Alizarin Red S stain confirmed the benefits of our cell block and demonstrated that such a novel combination and modification of biomaterials can serve as a platform for future clinical applications and use in dentin regeneration.

## 1. Introduction

According to the Centers for Disease Control and Prevention, 9 out of 10 adults over 18 years old have a certain degree of tooth caries [1]. Insults like these disrupt the process of dental root development and lead to the formation of necrotic immature permanent teeth. Current treatment approaches for such cases include disinfection of the root canal as well as subsequent induction of blood clots. Although this approach stimulates the repair and regeneration of dental pulp tissue, its regeneration process is difficult to control and may lead to suboptimal outcomes such as periodontal tissue ingrowth and incomplete revascularization. Therefore, novel treatment methodologies are still required to provide better endodontic regeneration [2]. Dental pulp tissue is a highly vascularized and innervated soft connective tissue that is critical to the development of dental roots [3]. It possesses mesenchymal stem cell-like properties, which include great proliferative, clonogenic, as well as multilineage differentiation capabilities [4,5,6]. Despite having these unique properties, it is greatly limited by its vulnerability to various insults such as caries, infections, and traumas. The emergence of stem cell-based study has gained a huge amount of attention in the field of regenerative dentistry. Human dental pulp stem cells (hDPSCs) have been widely used due to their unique potential of multilineage differentiation and their simple isolation method by pulp tissue outgrowth or enzymatic digestion [3,7,8]. 

With the emergence of tissue engineering technologies, scientists are now able to fabricate 3D cell spheroid cultures or cell blocks with various signaling cues to better mimic in vivo microenvironments and physiological functions of native tissues [9]. Cells in 3D culture were able to adopt proper structures, cellular interactions, and allow nutrient perfusion to all levels and thus are commonly used for modeling mechanisms of normal physiological processes, tumor biology, tissue regeneration, and monitoring of treatment efficacy [10,11]. Scientists are now able to combine different types of cells and signaling molecules in biomaterials with customizable mechanical properties to better mimic native structures [12]. Numerous types of biomaterials have been explored and considered for endodontic regeneration. Hydrogels, consisting of natural polymers such as gelatin and alginate, are commonly used in tissue engineering as they contain natural RGD motifs which have been proven to actively support cellular proliferation, migration, and differentiation [13]. Aldana et al. combined alginate with gelatin methacrylate (GelMa) so as to optimize both biological and physical characteristics of pure alginate or pure gelatin hydrogels for 3D printing [14]. Modification of gelatin with methacrylate allows photopolymerization of hydrogel, thus increasing mechanical and physical characteristics of hydrogel for different types of applications [15]. Countless studies have explored GelMa–alginate hydrogels for cell encapsulation in tissue regeneration applications and results have proven the possibility of GelMa for endodontic engineering. Ansari et al. encapsulated gingival mesenchymal stem cells and human bone marrow MSCs in GelMa–alginate hydrogels and showed that it is possible to guide stem cell differentiation by controlling hydrogel stiffness and providing biological cues [16].

A fundamental issue in tissue engineering is the availability of sufficient blood vessels for nutrient provision and removal of toxic waste [17]. Lee et al. provided a detailed systematic review into the roles of biomaterials in promoting angiogenesis for tissue regeneration [18]. Angiogenesis is a process whereby new blood vessels stem from existing blood vessels due to increased cytokines, chemokines, and most importantly, vascular endothelial growth factor (VEGF) [19]. The cytokines and chemokines lead to an increase in vascular permeability, thus causing extravasation of proteins and endothelial cells. The endothelial cells would then rapidly migrate to the regenerating or injured side and organize themselves into functional lumens connecting that area with an existing blood vessel. Various types of techniques have been developed to promote angiogenesis for tissue engineering such as controlling pore sizes, pore stiffness, surface topography, modification of biomaterials, and inclusion of endothelial cells or growth factors. Our previous results showed that inclusion of calcium silicate (CS) products into GelMa were able to enhance angiogenesis via increasing the secretion of VEGF from fibroblasts [20]. In fact, CS is the bioceramic that is always used as an endodontics material for root repair [21]. This was due to the sustained and gradual release of silicate (Si) and calcium (Ca) ions into the surrounding fluid upon degradation and immersion [22]. Si ions have been demonstrated to promote vascularization by inducing secretion of angiogenic-related factors from nearby fibroblasts [23]. Furthermore, the release of Si ions were also proven to regulate bone or tooth regeneration in various types of stem cells, notably hDPSCs [24,25]. Dashnyam et al. concluded that the release of appropriate Si ions, up to several ppm, into the surrounding fluid had several roles to play in increasing angiogenesis. Si ions were able to mimic hypoxic conditions, therefore increasing activation of downstream angiogenesis pathways such as VEGF and other proangiogenic factors [26]. The presence of proangiogenic factors would then increase stimulation of nitric oxide which in turn enhances vasodilation and angiogenesis. In addition, Si ions were reported to inhibit antiangiogenic factors and receptors such as RHD2 [27]. 

**Figure 1:**
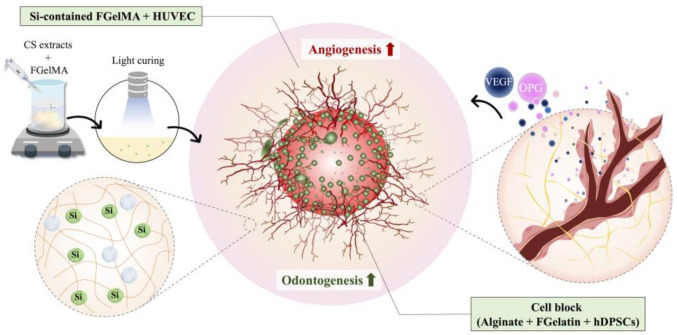
Schematic diagram of fabrication and hDPSCs-laden Alg/FGelMa cell block and HUVEC in Si-containing FGelMa hydrogels for endodontics application.

In this study, we fabricated alginate/fish gelatin (Alg/FGel) and CS-activated FGelMa cell blocks by applying the extract from CS to allow the dissolution of FGelMa. Then, both the FGelMa and Alg/FGel hydrogels were converted into stable cross-linked hydrogels by exposing FGelMa to UV light and by decreasing the temperature for Alg/FGel hydrogels, respectively. Figure 1 shows a schematic diagram of the fabrication process of the cell blocks. The concept of synergistic cell blocks with a co-culture of hDPSCs in Alg/FGel hydrogel and HUVEC in CS-activated FGelMa hydrogel was investigated. Cellular behavior was known to be greatly influenced by environmental factors. Therefore, the choice of materials used for the fabrication of scaffolds was an important key to successful regeneration of tissues. CS extracts addition into FGelMa hydrogels was proven to be advantageous for the angiogenesis of HUVEC by greatly enhancing the secretion of VEGF and OPG. Furthermore, Si ions were also able to promote angiogenesis indirectly via the mimicking of hypoxic conditions as well as inhibiting antiangiogenic factors which lead to the activation of subsequent angiogenic pathways. This enhancement of HUVEC angiogenesis in turn stimulates the proliferation and differentiation of hDPSCs through modulation of growth factors such as insulin-like growth factor as well as platelet-derived growth factors. In addition, co-culture studies using HUVEC and hDPSCs showed that both cell types were able to work synergistically with one another even without the aid of external signals or environmental cues. Therefore, in this study, we printed a synergistic cell block consisting of two different cell types in two modified light-curing biomaterials to further enhance endodontic regeneration by providing additional cues and mimicking the native microenvironment.

## 2. Materials and Methods

### 2.1. Preparation of the Extracts of CS Powders and Si-Contained FGelMa Hydrogels

CS were prepared according to our previous published methods [28]. In this study, 70% calcium oxide (CaO; Sigma-Aldrich, St. Louis, MO, USA) was mixed with 25% silicon dioxide (SiO_2_; Sigma-Aldrich, St. Louis, MO, USA) and 5% alumina oxide (Al_2_O_3_; Sigma-Aldrich, St. Louis, MO, USA) and sintered at 1400°C for 2 h. After cooling, the mixtures were placed into 99.5% ethanol and further ground with agate milling balls in a planetary ball mill machine (Retsch PM-100, Retsch GmbH, Hann, Germany) for 12 h. Then, the mixture was dried at 100 °C in an oven for 12 h. Extracts of the CS powders were first steam sterilized before immersion in tris buffer (25 mM, pH = 8.5). After stirring for 24 h, the mixtures were filtered, and the supernatants were sterilized using a 0.2 μm filter. The Si concentrations in the extracts were analyzed using inductively coupled plasma atomic emission spectrometry (ICP-AES; Perkin-Elmer OPT 1MA 3000DV, Shelton, CT, USA). 

Ten percent (w/v) gelatin from cold water fish skin (FGel, Sigma-Aldrich, St. Louis, MO, USA) was dissolved in 50°C phosphate buffer saline (PBS, Invitrogen, Grand Island, NY, USA). Then, methacrylate (Ma, Sigma-Aldrich, St. Louis, MO, USA) was added into the gelatin solution (Ma/gelatin ratio = 0.6) and left to react under stirring for 3 h. After the reaction, the mixture was centrifuged at 40°C and 8000 rpm to remove unreacted debris and dialyzed against deionized water using 10 kDa MWCO dialysis tubes (Thermo Fisher Scientific, Waltham, MA, USA). Purified FGelMa was then lyophilized for at least 72 h to obtain FGelMa sponge foams to be stored at −20°C. To prepare Si-FGelMa hydrogels, the FGelMa powder was first dissolved in CS extracts with varying concentrations of Si ions (Si0: 0 mM, Si0.5: 0.5 mM, and Si1.0: 1.0 mM) and stirred at 50°C for 10 minutes. Then, 0.25% of the lithium phenyl-2,4,6-trimethylbenzoylphosphinate photoinitiator (LAP, Sigma-Aldrich, St. Louis, MO, USA) was added to allow photo-curing. The various FGelMa hydrogels were then cured via exposed 365 nm UV light (Spot UV irradiation units Spot Cure Series, SP11, Ushio, Japan) for 90 s.

### 2.2. Alg/FGel Hydrogel Preparation for 3D Cell Block

A 15 mm square mold with a punched out 1 mm square at the base of the mold was used as a mold in this study. To fabricate the hydrogels, 20 g of FGel was first added into 80 mL of ddH_2_O to form a 20% FGel solution. The solution was then heated and stirred at 120°C for even dissolution of the FGel. The FGel solution was then sterilized, poured into the printed mold, and placed in a 4°C refrigerator to allow the FGel to solidify. Finally, 2.5% FGel and 1.2% Alg solution were mixed and sterilized prior to use. The Alg/FGel hydrogels were placed into the mold and cross-linked using 10 mM calcium chloride (CaCl_2_) in culture medium for 15 min in a 4 °C refrigerator. After which, the culture medium was replaced with a 9 mM CaCl_2_-containing medium and incubated in a 37 °C incubator for 2 h to sacrifice the mold to obtain a 3D block. 

### 2.3. Hydrogel Characterization

The microstructures of the Alg/FGel and FGelMa hydrogels were investigated under a cryo-scanning electron microscope (cryo-SEM, JSM-7800F, JEOL, Tokyo, Japan). In addition, the EZ-Test kit (Shimadzu, Kyoto, Japan) was used to obtain the stress–strain profile of the scaffold according to the ASTM D638 guidelines. The guideline stipulates the shape, size, and thickness of the scaffolds to be tested. The specimens were designed in the shape of a dumb-bell with the two ends clamped to the machine. A tensile test speed of 1 mm/s was applied from both ends until the scaffold was torn from the middle. Six scaffolds from each group were tested, and the experimental results were plotted with distance (mm) and load (N). In addition, ^1^H NMR (nuclear magnetic resonance) and Fourier-transform infrared spectroscopy (FTIR) was performed to evaluate the degree of substitution and bond formation. In short, the hydrogels were dissolved in deuterium oxide (Sigma-Aldrich, St. Louis, MO, USA) and analyzed using a Bruker AVANCE 500 MHz (Bruker BioSpin, Rheinstetten, Germany). An FTIR (Vertex 80v, Bruker, Karlsruhe, Germany) was used to assess the functional groups of the hydrogels from 500 to 3000 cm^−1^.

### 2.4. Weight Loss/Degradation and Release Profile of the Hydrogels

For this study, the various Si-containing FGelMa composited were immersed in simulated body fluid (SBF) to evaluate the weight loss and Si release profiles of the scaffolds over 14 days. The composition of SBF used in this study is similar to that of human plasma, and its formulated compounds include 7.9949 g NaCl, 0.2235 g KCl, 0.147 g K_2_HPO_4_, 0.3528 g NaHCO_3_, 0.071 g Na_2_SO_4_, 0.2775 g CaCl_2_, and 0.305 g MgCl_2_·6H_2_O. These compounds were dissolved in 1000 mL of distilled water in order, and tris buffer and HCl were added at the end to adjust the pH to 7.4. The scaffolds from each group were soaked in 37°C SBF for a fixed period of time, and the degradation rate was calculated using the following formula:

Degradation rate (%) = (W_0_ − W_t_)/W_0_ × 100%

In addition, inductively coupled plasma atomic emission spectroscopy (ICP-AES, Perkin-Elmer OPT 1MA 3000DV, Shelton, CT, USA) was used to measure for the amount of Si ions released after periods of immersion.

### 2.5. In Vitro Biological Tests

In this study, we used hDPSCs (Lonza, PT-5025, Lonza, Basel, Switzerland) and HUVEC (ScienCell Research Laboratories, Carlsbad, CA, USA) for the co-cultivation study and they were cultured in different bioinks. Therefore, we first evaluated the effects of different bioinks on cells, and both used staining and PrestoBlue to evaluate the biocompatibility. Firstly, 1.0 × 10^7^/mL of hDPSCs were encapsulated in Alg/FGel hydrogel to form the core of our cell block. The hDPSCs were cultured to passage 4–7 in a 37°C humidified atmosphere with 5% CO_2_. After 1, 3, and 7 days of culture, live/dead assay (Invitrogen, Carlsbad, CA, USA) and PrestoBlue assay (Invitrogen, Carlsbad, CA, USA) were used to quantify cell survival rate. The live/dead assay kit was used to conduct fluorescent staining observed using the confocal spectral microscope (Leica TCS SP8, Wetzlar, Germany). The fluorescent staining method marked the live cell membrane green and the dead cell membrane red, respectively. In addition, the cell blocks were reacted with PrestoBlue reagent at a ratio of 9:1, and then added to the culture well for 120 min at 37 °C incubator. Next, 100 μL of the solution was transferred to a new 96-well plate. The optical density of the solution was assessed at a wavelength of 570 nm (reference wavelength of 600 nm) using a spectrophotometer (Infinite Pro M200, Tecan, Männedorf, Switzerland). All tests were performed in triplicate. Then, 6.0 × 10^7^/mL of HUVEC were encapsulated in Si-FGelMa to form the periphery of our cell block. After culture for different time-points, the constructs were fixed with 4% paraformaldehyde (Sigma-Aldrich, St. Louis, MO, USA) for 30 min at room temperature. The constructs were permeabilized with 0.1% Triton X-100 (Sigma-Aldrich, St. Louis, MO, USA) in PBS for 20 min. The F-actin cytoskeleton was stained with fluorescent dye (Alexa Fluor 488, Invitrogen, Carlsbad, CA, USA) conjugated with phalloidin according to the manufacturer’s instructions. In addition, 300 nM of 4’,6-diamidino-2-phenylindole (DAPI, Invitrogen, Carlsbad, CA, USA) was used to stain the cell nuclei. Then, the cell morphology and cell distribution were observed using a confocal microscope (Leica Microsystems GmbH, Wetzlar, Hessen, Germany). All tests were performed in triplicate. Finally, we also used the PrestoBlue to consider the quantity of HUVEC survival rate according to the methods described above.

### 2.6. Western Blot

After 3 days of culture, HUVEC were lysed using Nonidet-P40 buffer (NP40, Sigma-Aldrich, St. Louis, MO, USA), and a BCA protein assay kit (Thermo Fisher Scientific, Waltham, MA, USA) was used to assess for the protein concentrations of different Si-containing FGelMa hydrogels. Sodium dodecyl sulfate (SDS)–polyacrylamide gel electrophoresis was used to segregate the cell lysates (40 μg protein), which were then transferred onto polyvinylidene difluoride (PVDF) membranes (Merck Millipore, Burlington, MA, USA). These were placed in a solution containing 2% bovine serum albumin and tris-buffered saline with Tween-20 for 1 h before they were exposed to primary anti-Angiopoietin 1 (Ang1, ABclonal, Woburn, MA, USA), anti-Angiopoietin 2 (Ang2, ABclonal, Woburn, MA, USA), anti-von Willebrand factor (vWF, Abcam, Cambridge, MA, USA), and β-actin (Abcam, Cambridge, MA, USA) for 2 h for further immunoblotting. Thereafter, horseradish peroxidase (HRP)-conjugated secondary antibodies were incubated with the samples for 1 h to enable chemiluminescence visualization. For this study, protein expression levels were normalized to β-actin. All experiments were performed in triplicate.

### 2.7. Enzyme-Linked Immunosorbent Assay (ELISA)

An ELISA was used to analyze the levels of vascular endothelial growth factor (VEGF, KHG0111, Invitrogen, Carlsbad, CA, USA) and osteoprotegerin (OPG, ab100617, Abcam, Cambridge, MA, USA) in the medium of the HUVEC-laden Si-FGelMa hydrogels after a specified duration of culture. These ELISA kits were used according to the manufacturer’s instructions and were further correlated to a standard curve. All the cultivation runs were performed three times in the experiments.

### 2.8. hDPSCs and HUVEC Cell Co-Culture Analysis

In order to clarify the interaction between 3D hDPSCs-laden Alg/FGel and the HUVEC-containing Si-FGelMa hydrogel, we first observed the cell morphology. First, hDPSCs and HUVEC were treated with CellTracker™ CM-Dil Dye (Invitrogen, Carlsbad, CA, USA) and CellTracker™ Green CMFDA Dye (Invitrogen, Carlsbad, CA, USA) for 30 min, and washed with PBS several times before testing. According to the previous method, hDPSCs with red fluorescence and Alg/FGel can be prepared into spheres. After mixing hDPSCs-laden Alg/FGel spheres with HUVEC-containing FGelMa, they were cured with UV light in accordance with the previous method. Finally, the constructs containing hDPSCs and HUVEC were cultured in the commercial medium and changed once every two days. Finally, the cell morphology and cell distribution were cultured for different days and observed using a confocal microscope (Leica Microsystems GmbH, Wetzlar, Hessen, Germany).

### 2.9. Odontogenesis Differentiation Assay

In order to evaluate the effect of different external factors on odontogenesis, the 3D hDPSCs blocks were loaded in HUVEC-laden Si-containing FGelMa hydrogels. These 3D constructs were cultured in an osteogenic differentiation culture medium (StemPro^TM^ osteogenesis differentiation kit, Invitrogen, Carlsbad, CA, USA) for 3 and 7 days. Then, NP40 Cell Lysis Solution (Sigma-Aldrich, St. Louis, MO, USA) was added to the scaffolds and centrifuged at 6000 rpm for 15 min for cell lysis, after which pNPP (Sigma-Aldrich, St. Louis, MO, USA) was added, and the absorbance was assessed using a 405 nm wavelength spectrophotometer to measure the alkaline phosphatase (ALP) level. The measured absorbance was standardized with the protein quantitative detection reagents (BCA, Thermo Fisher Scientific, Waltham, MA, USA). In addition, the production of dentin matrix protein-1 (DMP-1, MBS167298, MyBioSource, San Diego, CA, USA) and osteocalcin (OC, ab195214, Abcam, Cambridge, MA, USA) protein secretion from the hDPSCs were determined using these ELISA kits, following the manufacturer’s instructions. The protein concentrations were measured based on correlations with a standard curve. All experiments were performed in triplicate.

### 2.10. Mineralization

The formation of mineralized nodules of 3D hDPSCs were measured after 7 and 14 days of culture. Firstly, the cells were fixed with 4% paraformaldehyde (Sigma-Aldrich, St. Louis, MO, USA) for 15 min and then stained with 0.5% Alizarin Red S (Sigma-Aldrich, St. Louis, MO, USA) for 30 min at pH 4.2. A confocal microscope was utilized to observe the mineralization cells in the dark state. 

### 2.11. Statistical Analysis

A one-way variance statistical analysis and Scheffe’s multiple comparison test were used in this study to evaluate for differences between each group and scaffold. A value of <0.05 was considered to be statistically significant.

## 3. Results and Discussion

### 3.1. Synthesis and Characterization of the FGelMa and Alg/Gel Hydrogels

**Figure 2:**
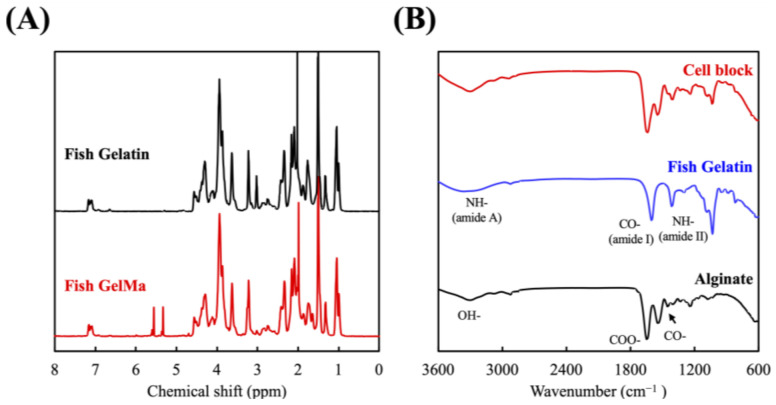
(**A**) The ^1^H NMR spectra of FGel and FGelMa and (**B**) Fourier-transform infrared spectroscopy results for Alg, FGel, and Alg/FGel (cell block).

The degrees of functionalization and chemical bonds were analyzed using 1H NMR and FTIR and the results are shown in Figure 2. The conjugation of the methacrylate groups to FGel was confirmed in Figure 2A by the presence of methacrylate vinyl groups at δ = 5.4 and 5.7 ppm. In addition, there was a decrease of signal at δ = 2.9 ppm which stands for the protons of methylene of lysine, thus further indicating the successful conjugation of methacrylate to FGel [29]. It was also important to note that the modification did not alter the original structural characteristics of gelatin, therefore allowing us to retain its RGD sequences which were favorable for cellular proliferation while achieving enhanced mechanical properties simultaneously [30]. FTIR was used for the identification of various amino acids and bonds in the cell block and results were shown in Figure 2B. The FTIR absorption range of FGel falls within the amide band region which represents various vibrational modes of the peptide bone. Amide-I represents C=O stretching/hydrogen bonding coupled with COO and amide-II represents bending vibration of N–H groups and stretching vibrations of C–N groups [31]. As seen, the cell block had shown the C=O and N–H peaks at 1650 and 1300 cm^−1^ of FGel and the C=O and COO peaks at 1790 and 1400 cm^−1^ of Alg, respectively [32]. Similarly, these results clearly indicated that cell blocks consisting of both gelatin and alginate could be fabricated using our method as described above.

**Figure 3:**
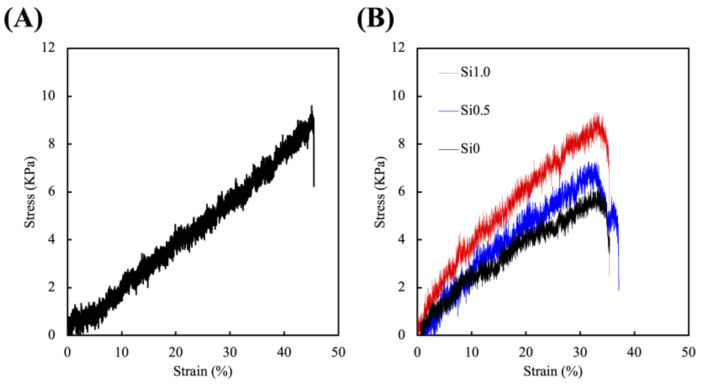
Stress–strain curve of (**A**) Alg/FGel hydrogel and (**B**) Si-containing FGelMa hydrogel.

The stress–strain curves of Alg/FGel and Si-FGelMa specimens were as reported in Figure 3. All parameters including duration, distance, and intensity of UV exposure was kept constant throughout this test and were conducted on dumb-bell-shaped specimens of the hydrogels. The Alg/FGel showed the mechanical properties of approximately 9 kPa (Figure 3A). The mechanical property of a hydrogel is an important factor as it is known to influence cellular morphology, which in turn affects cellular functions and behaviors [33]. Stiffer hydrogels were known to enhance cytoskeletal organization, thus increasing osteoblastic/odontogenic differentiation and proliferation of stem cells [34]. Furthermore, numerous results had proved that alginate coupled with RGD motifs such as gelatin significantly enhanced cellular adhesion, migration, proliferation, and differentiation as alginate itself does not promote cellular activities due to the lack of cellular adhesion motifs [35]. In addition, the increasing Si concentration had a positive impact on the mechanical properties of the FGelMa-based hydrogels (Figure 3B). As shown, increasing the concentrations of Si positively regulated the stress, with Si0.5 and Si1.0 increasing the stress by 30% and 69% than Si0, respectively. As reported previously, the addition of Si ions increased Si–OH bonds, thus increasing the overall mechanical properties of the hydrogels [20]. According to the rheological and mechanical test, Yu et al. demonstrated that the addition of CS material could significantly regulate the printability compared to FGelMa alone, and with the increased concentration of CS material, the mechanical properties increased [36]. In addition, increasing mechanical properties of the hydrogels also allows better surgical handling during implantations.

### 3.2. Effects of Degradation Profiles in the Immersion Experiments

**Figure 4:**
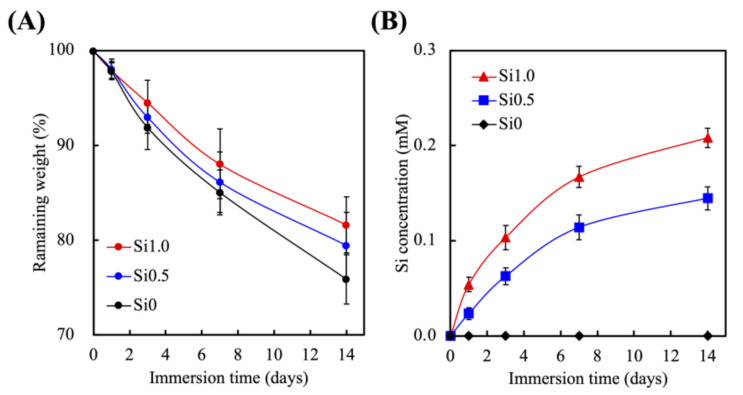
(**A**) The weight loss and (**B**) Si ions released from the Si-containing FGelMa hydrogels after soaking in SBF for 1, 3, 7, and 14 days. Data presented as mean ± SEM, n = 6 for each group.

The degradation rate and Si release profiles of the Si-FGelMa were conducted over 14 days of immersion in SBF and as shown in Figure 4. They experienced rapid degradation during the first 2 days of immersion with Si0, Si0.5, and Si1 having lost 9%, 8%, and 4% of their total weight (Figure 4A). After which, the degradation rates slowed to a gradual pace till 14 days of immersion. After 14 days of immersion, Si0, Si05, and Si1 had 83%, 81%, and 75% of their weight remaining, respectively. As specified above, FGelMa-based hydrogel was noted to have fewer amino acids as compared to gelatin, thus resulting in lower mechanical properties and higher swelling behaviors which leads to higher degradant properties [37]. In the Si-containing FGelMa, we consider that the reason for the lower degradation rate may be in the light-cured FGelMa hydrogels because the addition of Si extract regulated the curing and hydration process and improves the ionic bonding between the FGelMa matrix that inhibited the degradation rate. Furthermore, it could be noted that the Si-FGelMa hydrogels had controllable and tunable degradation rates according to the concentrations of Si, thus making it an even more appealing biomaterial for endodontic regeneration [38]. In Figure 4B, the increasing of Si concentrations in FGelMa hydrogels led to a gradual increase in the release of Si ions during the immersion period. After immersion for 14 days, the Si concentrations of SBF were approximately at 0.15 ± 0.02 mM and 0.21 ± 0.01 mM for Si0.5 and Si1.0. The Si ions were only present in Si-containing FGelMa hydrogels; therefore, the levels of Si ions for CS0 were 0 mM at all time-points. Similar to the degradation profiles, Si0.5 and Si1.0 groups showed the burst-type release of Si ions into SBF for the first 3 days, after which the release of Si ions gradually stabilized. After 14 days of soaking, the Si-containing FGelMa specimens were releasing Si ions that supported other important factors to observe that the profiles of Si ions released could be regular with the rate of tissue regeneration [39]. In addition, Liao et al. demonstrated the carriage of Si ions alone activated osteogenesis differentiation of bone marrow-derived mesenchymal stem cells in the absence of osteogenic-inducing factors [40]. Taken together, these results proved that the addition of Si ions into FGelMa hydrogels not only improved the mechanical behaviors and degradation profiles but also released an adequate concentration of Si ions, which is expected to assist in the endodontic regeneration.

### 3.3. In Vitro hDPSCs Culture

**Figure 5:**
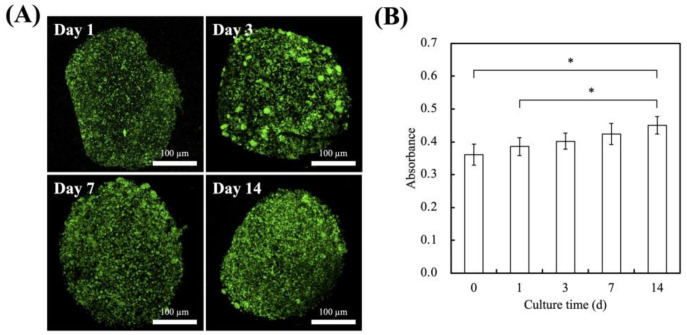
(**A**) Live/dead staining and (**B**) cell viability of hDPSCs-laden Alg/FGel cell block for various time-points. Scale bar = 100 µm. (Green: live cells; red: dead cells.) * Indicates a significant difference (*p* < 0.05). Data presented as mean ± SEM, n = 6 for each group.

The live/dead staining and cytotoxicity assay of the hDPSCs were evaluated over 14 days, and the results are shown in Figure 5. As seen from Figure 5A, there was an obvious increase in the number of green fluorescence staining on day 3, 7, and 14 as compared to day 1. After 14 days of culture, hDPSCs occupied a wider surface area and were more clustered as compared to day 1, 3, and 7. Similarly for the quantification results of cytotoxicity in Figure 5B, there were significantly higher levels of cellular proliferation of 1.2 and 1.3 times at day 14 as compared to day 0 and day 1, respectively. Alginate and gelatin are both common biomaterials used in hard tissue regeneration and cell carriers [41,42,43]. However, alginate does not promote cellular adhesion and attachment, thus leading to poor cell–material interactions. In addition, alginate has a slow and uncontrolled degradation rate as it is not enzymatically degradable in mammals. Therefore, gelatin, a natural derivative of collagen, is often added into alginate hydrogels to improve bioactivity as it contains an abundance of RGD motifs for cellular adhesion and proliferation. The main disadvantage of gelatin lies in its poor mechanical properties; however, this issue is resolved with the combination of alginate.

### 3.4. In Vitro HUVEC Culture

**Figure 6:**
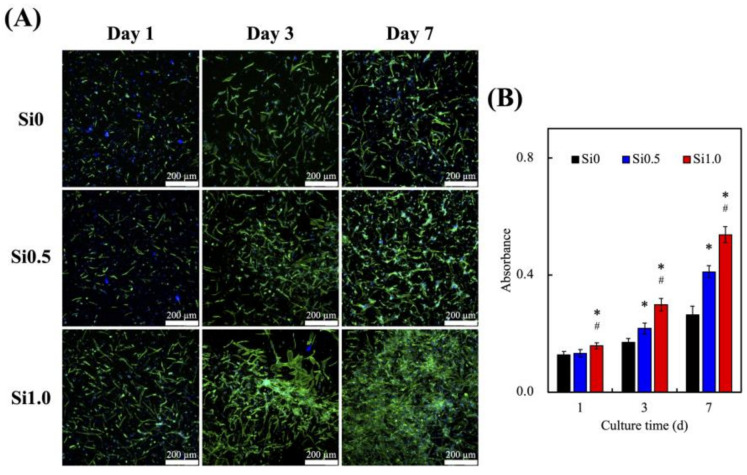
(**A**) F-actin (green)/DAPI (blue) staining and (**B**) proliferation rate of HUVEC in various Si-containing FGelMa hydrogels for different days. * Indicates a significant difference (*p* < 0.05) from Si0. # Indicates a significant difference (*p* < 0.05) from Si0.5. Data presented as mean ± SEM, n = 6 for each group. Scale bar = 200 µm.

In order to further understand the effect of Si ions on angiogenesis of HUVEC and what cytokine secretion is promoted to enhance the effect of hDPSCs in odontogenesis, we evaluated cell proliferation, and the production and secretion of various related proteins. As shown in Figure 6A, higher cellular proliferation was observed in both Si0.5 and Si1.0 groups on the first day of culture as compared to those in Si0. In addition, the cells exhibit clear and sharp morphology with wide extensions of filopodia, indicating that Si-FGelMa hydrogels were able to provide an optimal 3D microenvironment for the growth of HUVEC. A significantly higher number of cells with homogeneous distributions were observed in the Si1.0 group on day 7 of culture as compared to the other two groups that also supported the enhancement of cellular adhesions and intercellular interactions, leading to further formation of connections and growth. When HUVEC shows a network structure, it can also be inferred that a higher proportion can undergo angiogenesis [44]. Then, the biocompatibility of HUVEC-laden Si-containing FGelMa hydrogels were evaluated using PrestoBlue assays (Figure 6B). The absorbance level of the Si1.0 group on day 7 of culture showed a 2-fold increase as compared to those in the Si0 group. In addition, the Si0.5 group also exhibits a similar growth pattern as the Si1.0 group, as evidenced by the increasing levels of absorbance with longer days of culture. These results suggest the Si-containing FGelMa hydrogels released suitable Si concentrations within the range of 0.14–0.20 mM in the medium that was able to regulate growth and proliferation of HUVEC. Similarly, several studies have been indicated affirming that the initial levels of cell adhesion can be related to predict consequent cellular functions, and they could be used as a positive indication for cell behaviors, such as proliferation and differentiation [45,46]. Noteworthily, Si1.0 maybe has the optimal concentration of Si ions for HUVEC growth and maturation, which assist the potential sequent differentiation of hDPSCs in the later stage.

**Figure 7:**
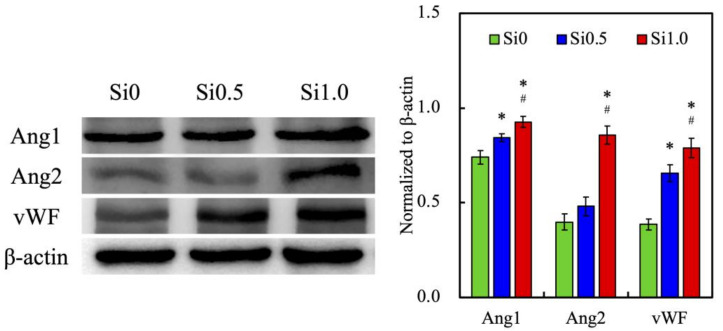
The in vitro effects of angiogenesis-related markers (Ang1, Ang2, and vWF) of HUVEC-laden Si-containing FGelMa hydrogels. * Indicates a significant difference (*p* < 0.05) from Si0. # Indicates a significant difference (*p* < 0.05) from Si0.5. Data presented as mean ± SEM, n = 3 for each group. Scale bar = 200 µm.

In order to confirm whether the Si ions in the FGelMa hydrogel can enhance the angiogenesis ability of HUVEC, we used a Western blot for the evaluation of various angiogenic proteins such as Angiopoietin-1 (Ang1), Angiopoietin-2 (Ang2), and von Willebrand factor (vWF) as shown in Figure 7. After being cultured for 3 days, HUVEC showed higher expression levels of Ang1, Ang2, and vWF that were observed in both Si0.5 and Si1.0 as compared to Si0. The significant increase in expression levels of Ang1, Ang2, and vWF were prominent in the Si1.0 and 1.25, 2.16, and 2.05 times of that observed in the Si0, respectively. These results demonstrated that the release of Si ions from FGelMa not only promoted HUVEC growth, but also regulated the downstream angiogenic effects of HUVEC that were directly related to the concentration of Si ions in FGelMa hydrogels. Ang1 and Ang2 are important proteins that mediate angiogenesis through their agonist and antagonistic interactions with VEGF [47,48]. On the other hand, vWF was an important factor in the regulation of the formation of thrombus and participates in the coagulation pathway [49]. Angiogenesis is a key factor to successful hard tissue regeneration and HUVEC are always reported to possess angiogenic effects [50]. In a previous study, we proved that hDPSCs alone treated with CS were able to enhance vWF and Ang1 expressions, thus subsequently increasing angiogenesis [44]. Hence, we can conclude that Si-containing FGelMa hydrogel was one of the key components in supporting the cell function of HUVEC and was able to produce a synergistic effect in odontogenesis for regeneration.

**Figure 8:**
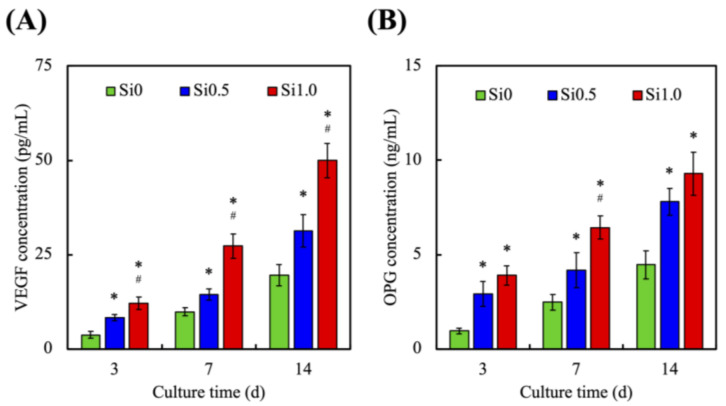
(**A**) VEGF and (B) OPG secreted form HUVEC in various Si-containing FGelMa hydrogels for different times. * Indicates a significant difference (*p* < 0.05) from Si0. # Indicates a significant difference (*p* < 0.05) from Si0.5. Data presented as mean ± SEM, n = 6 for each group.

In the past, many studies have confirmed that the cytokines released during angiogenesis can effectively promote the differentiation of surrounding cells [51]. However, the crosstalk between hDPSCs and HUVEC can direct the differentiation of stem cells toward stem cells that later wrap osteo-/odontogenesis to form functional hard tissue [52]. In this study, we have found out that our cell-laden bioinks can enhance odontogenesis and angiogenesis to a further extent. Figure 8 shows that the HUVEC secreted a significantly greater amount of VEGF (Figure 8A) and OPG (Figure 8B) proteins in the Si1.0 group. In addition, OPG concentrations in Si0.5 and Si1.0 were 1.7 and 2.1 times higher than Si0 after 14 days of cell culturing; similarly, VEGF was at least 1.5-fold of Si0 after the same length of culture time. From the data, we found out that Si0 hydrogel simulates the very low secretion of VEGF and OPG. The increase of VEGF may be mainly due to the presence of HUVEC in Si1.0 hydrogel. Therefore, the expression of OPG biomarkers in the Si1.0 hydrogel was also greater than other groups. The endothelial cells play an important role in the interaction between bone regeneration and angiogenic differentiation [53]. During the incubation period, HUVEC populating in hydrogel exhibited a satisfying growth status, as expected. Analysis of human tissues show that endothelial cells in normal surrounding tissue express OPG. Moreover, HUVEC and other endothelial cells both expressed OPG during angiogenesis [51]. Therefore, the above results not only indicate that Si plays a role in enhancing angiogenesis, but also stimulate HUVEC to secrete a variety of cytokines that can assist odontogenic differentiation, which is beneficial to endodontic regeneration engineering.

**Figure 9:**
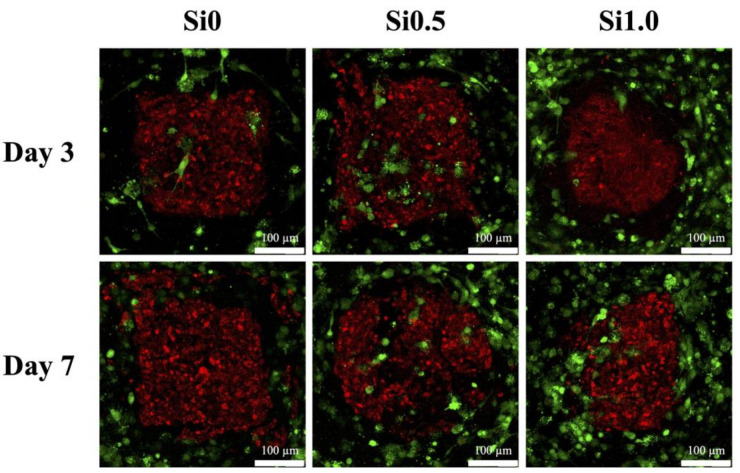
The staining of hDPSCs cell block (red) and HUVEC (green) co-cultured in various Si-containing FGelMa hydrogels for 3 and 7 days.

Both HUVEC and hDPSCs were immunofluorescence-stained with red and green fluorescence, respectively, and are shown in Figure 9. As seen, the hDPSCs were homogeneously encapsulated in Alg/FGel in the middle with HUVEC at the perimeters. Most importantly, HUVEC in the Si1.0 hydrogel started to proliferate and migrate from the perimeters towards the core of the hDPSCs clusters. On the other hand, after 7 days of culture, there was limited proliferation of HUVEC in the Si0 hydrogel. Jin et al. co-cultured hDPSCs with HUVEC and showed that such an ecosystem improved expressions of osteogenic and angiogenic genes [54]. In this study, we took a step further in providing a more suitable microenvironment with stimulating factors for the various types of cells. HUVEC were known to proliferate and migrate from main branches located at the periphery towards the center of the regenerating tissues. Therefore, HUVEC were purposely placed at the perimeter of the cell block and results showed that the addition of Si-containing hydrogels not only provided HUVEC with enhanced angiogenic-related protein expression but also guided them to form tubular-like structures.

### 3.5. Odontogenic Behaviors

**Figure 10:**
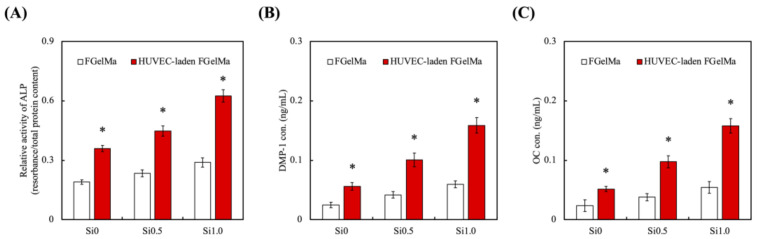
Odontogenesis-related differentiation markers of (**A**) ALP activity, (**B**) DMP-1, and (**C**) OC expression of hDPSCs-laden Alg/FGel cell block in Si-containing FGelMa/HUVEC-laden FGelMa hydrogels and cultured for 7 days. * Indicates a significant difference (*p* < 0.05) from FGelMa. Data presented as mean ± SEM, n = 6 for each group.

**Figure 11:**
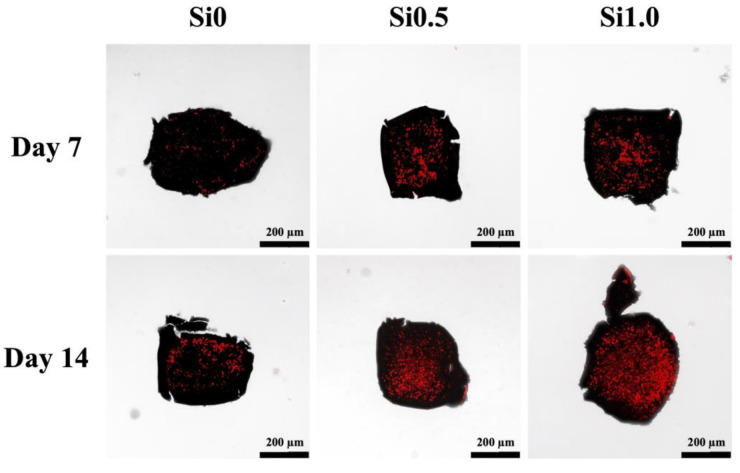
Alizarin Red S staining of hDPSCs-laden Alg/FGel cell block in various HUVEC-containing FGelMa for 7 and 14 days. The scale bar is 200 μm.

In order to assess the feasibility of hDPSCs-laden Alg/FGel under various concentrations of Si-containing FGelMa with or without co-culture of HUVEC, quantification of odontogenesis-related markers including alkaline phosphatase (ALP, Figure 10A), dentin matrix phosphoprotein 1 (DMP-1, Figure 10B), and osteocalcin (OC, Figure 10C) were evaluated using the ELISA. Significantly increased levels of ALP, DMP1, and OC were observed on hDPSCs cultured with the addition of HUVEC-laden FGelMa than FGelMa only. In addition, the increases were proportional to the amount of Si-containing FGelMa hydrogel; Si0.5 and Si1.0 had enhanced significantly 25% and 74% higher levels of ALP as compared to Si0. Similar trends were noted for DMP-1 and OC with Si1.0 having 2.66 and 2.91 times higher levels, respectively, as compared to FGelMa alone. In a previous study, Si has also been demonstrated to improve odontogenesis/angiogenesis-related proteins that regulated collagens and non-collagenous proteins such as glycoproteins and proteoglycans of which, ALP, DMP-1, and OC make up the bulk of the glycoproteins group [55]. Previously, we had elaborated on the influence of HUVEC on hDPSCs in dentin regeneration. HUVEC are known to secrete endothelin-1 and insulin-like growth factor that induces and activates hDPSCs proliferation and differentiation. Recently, scientists discovered that hDPSCs expressed CD146 markers, thus suggesting hDPSCs might be of peri-vascular origin. Scientists further discovered that migration of pre-odontoblasts to blood vessels might be due to by-products released from the breakdown of dentin. The regeneration of dentin is then therefore regulated by HUVEC as elaborated above. Therefore, the results above further confirmed that addition of HUVEC and Si ions enhanced secretion of angiogenic and odontogenic factors. Finally, the Alizarin Red S staining was used to identify the presence of calcium and is shown in Figure 11. Calcium reacts with Alizarin Red S and forms a complex via a chelation process to form a bright red stain. It is important to note that mineralization usually occurs during the final stages of odontogenesis. In addition, due to high concentration of hDPSCs encapsulated into the hydrogels, the confocal images would appear with a dark background. At day 7, significant calcium deposits were noted as numerous mineralized nodules in the Si1.0 and Si0.5 groups. Little to no calcium deposits were noted in the Si0 group at day 7 of culture. The amount of calcium deposits greatly increased after 14 days of culture as seen from the increased Alizarin Red S staining [56]. Taking the results discussed previously into account, it is reasonable to conclude that the Si-containing FGelMa hydrogel not only up-regulates the expression of angiogenesis-related markers of HUVEC but also mediates the improving of hDPSCs odontogenic differentiation of hDPSCs. Numerous studies have shown that co-culture of stem cells enhanced regeneration capabilities of hydrogels, regardless of whether it is in the wound healing, orthodontic, orthopedic, or soft tissue regeneration. There is definitely a need to attempt to replicate the synergistic communications and interactions between the microenvironment and various cells present in native tissues in order to bring about efficient tissue regeneration. Of which, angiogenesis is another factor that is critical for tissue regeneration. Currently, there are limited in vivo tissue engineering studies exploring the effects of HUVEC in tissue engineering. However, there are in vivo clinical trials available for the application of HUVEC vaccination for certain diseases such as glioblastoma. Results have shown that patients with the HUVEC vaccination had a more controlled and stable disease as compared to those with no vaccinations. Even though the specialty of stem cell treatment is still generally in its infant stages, we firmly believe that the application of stem cells will be the way ahead for tissue regeneration and tissue engineering.

## 4. Conclusions

Dental caries is a common dental disease affecting billions of people worldwide. Tissue engineering has since gained traction for the treatment of various clinical issues caused by dental caries. In this study, we fabricated cell blocks using hDPSCs at the core with Alg/FGel hydrogel that encased with Si-containing FGelMa hydrogel with HUVEC. This design attempted to mimic the native microenvironment of regenerating endodontic-related tissues with angiogenesis migrating from the periphery to support regeneration. The capability to release Si ions increased mimicry of the environment and thus activation of various angiogenic downstream signals, thus leading to increased expressions and secretions of angiogenesis-related markers. In addition, the design of hDPSCs cell blocks allowed for crosstalk with HUVEC and taken together, enhances odontogenic-related marker expression. Subsequent Alizarin Red S stains confirmed the benefits of our cell block and demonstrated that such a novel combination and modification of biomaterials can serve as a platform for future clinical applications and use in endodontic regeneration. From this study, it was found that Si-containing FGelMa and Alg/FGel hydrogels had good potential as an alternative to current endodontic regeneration strategies.

## Data Availability

Data available in a publicly accessible repository.
